# Fibroblast‐specific genome‐scale modelling predicts an imbalance in amino acid metabolism in Refsum disease

**DOI:** 10.1111/febs.15292

**Published:** 2020-03-31

**Authors:** Agnieszka B. Wegrzyn, Katharina Herzog, Albert Gerding, Marcel Kwiatkowski, Justina C. Wolters, Amalia M. Dolga, Alida E. M. van Lint, Ronald J. A. Wanders, Hans R. Waterham, Barbara M. Bakker

**Affiliations:** ^1^ Systems Medicine of Metabolism and Signalling Laboratory of Paediatrics University of Groningen University Medical Centre Groningen The Netherlands; ^2^ Analytical Biosciences and Metabolomics Division of Systems Biomedicine and Pharmacology Leiden Academic Centre for Drug Research Leiden University The Netherlands; ^3^ Laboratory Genetic Metabolic Diseases Department of Clinical Chemistry Amsterdam UMC, Location AMC University of Amsterdam The Netherlands; ^4^ Centre for Analysis and Synthesis Department of Chemistry Lund University Sweden; ^5^ Department of Laboratory Medicine University of Groningen University Medical Center Groningen The Netherlands; ^6^ Pharmacokinetics, Toxicology and Targeting Groningen Research Institute of Pharmacy (GRIP) University of Groningen The Netherlands; ^7^ Mass Spectrometric Proteomics and Metabolomics Institute of Biochemistry University of Innsbruck Austria; ^8^ Laboratory of Paediatrics University Medical Centre Groningen University of Groningen The Netherlands; ^9^ Department of Molecular Pharmacology Groningen Research Institute of Pharmacy University of Groningen The Netherlands

**Keywords:** amino acids, fibroblast, genome‐scale modelling, metabolism, Refsum disease

## Abstract

Refsum disease (RD) is an inborn error of metabolism that is characterised by a defect in peroxisomal α‐oxidation of the branched‐chain fatty acid phytanic acid. The disorder presents with late‐onset progressive retinitis pigmentosa and polyneuropathy and can be diagnosed biochemically by elevated levels of phytanate in plasma and tissues of patients. To date, no cure exists for RD, but phytanate levels in patients can be reduced by plasmapheresis and a strict diet. In this study, we reconstructed a fibroblast‐specific genome‐scale model based on the recently published, FAD‐curated model, based on Recon3D reconstruction. We used transcriptomics (available via GEO database with identifier GSE138379), metabolomics and proteomics (available via ProteomeXchange with identifier PXD015518) data, which we obtained from healthy controls and RD patient fibroblasts incubated with phytol, a precursor of phytanic acid. Our model correctly represents the metabolism of phytanate and displays fibroblast‐specific metabolic functions. Using this model, we investigated the metabolic phenotype of RD at the genome scale, and we studied the effect of phytanate on cell metabolism. We identified 53 metabolites that were predicted to discriminate between healthy and RD patients, several of which with a link to amino acid metabolism. Ultimately, these insights in metabolic changes may provide leads for pathophysiology and therapy.

**Databases:**

Transcriptomics data are available via GEO database with identifier GSE138379, and proteomics data are available via ProteomeXchange with identifier PXD015518.

## Graphical abstract

Refsum disease (RD) is an inborn error of metabolism that is characterised by a defect in peroxisomal α‐oxidation of the branched‐chain fatty acid phytanic acid. In this study, we used various omics obtained from healthy controls and RD patient fibroblasts to reconstruct a fibroblast‐specific genome‐scale model. Using this model, we identified 53 metabolites that discriminated between healthy and RD patients, several of which with a link to amino acid metabolism.

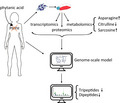

**Abbreviations**
3‐MAA: 3‐methyladipate
4,8‐DMN‐CoA: 4,8‐dimethylnonanoyl‐CoA
CTRL: control
CYP: cytochrome P450 family
PCA: principal component analysis
PHYH: phytanoyl‐CoA 2‐hydroxylase
RD: Refsum disease

## Introduction

Peroxisomes are organelles that, among other functions, are crucial for cellular lipid metabolism. They perform both anabolic and catabolic processes, including the α‐ and β‐oxidation of very‐long‐chain fatty acids, dicarboxylic acids and methyl‐branched‐chain fatty acids [1]. Furthermore, peroxisomes are involved in the biosynthesis of ether phospholipids, including plasmalogens, bile acids and essential polyunsaturated fatty acids such as docosahexaenoic acid [2].

Refsum disease (RD) is a rare inborn error of peroxisomal metabolism with an unknown incidence. It probably remains highly unrecognised since the awareness of inborn errors of metabolism is low among ophthalmologists. RD is caused by biallelic mutations in the gene encoding phytanoyl‐CoA 2‐hydroxylase (PHYH), resulting in defective α‐oxidation of the branched‐chain fatty acid phytanate (3,7,11,15‐tetramethylhexadecanoate) [3]. Phytanate contains a 3‐methyl group and is therefore not a substrate for peroxisomal β‐oxidation. Consequently, phytanate first needs to undergo α‐oxidation, thereby producing pristanate, which then can be further degraded by β‐oxidation [2]. An alternative metabolic pathway for the breakdown of phytanate is ω‐oxidation, which takes place in the endoplasmic reticulum [4]. The end product of ω‐oxidation of phytanate is 3‐methyladipic acid (3‐MAA), and ω‐oxidation has been described to be upregulated in patients with RD [5]. RD was first described in 1945 and is clinically characterised by progressive retinitis pigmentosa, polyneuropathy, cerebellar ataxia and deafness [5]. Biochemically, RD is diagnosed by elevated levels of phytanate in plasma and tissues. Phytanate solely derives from the diet, and patients with RD are mostly diagnosed in late childhood [3, 5]. To date, patient management focuses on the reduction of phytanate levels by plasmapheresis and a strict diet to reduce the intake of dairy‐derived fat [6].

Recently, computational models have become valuable tools to study the complex behaviour of metabolic networks. One type of computational models is genome‐scale models of metabolism, which contain all currently known stoichiometric information of metabolic reactions, together with enzyme and pathway annotation [7]. These models can further be constrained and validated by incorporation of different types of data, including mRNA and metabolite profiles, as well as biochemical and phenotypic information [8]. To date, the most comprehensive human models are Recon3D [9] and HMR 2.0 [10], which are consensus metabolic reconstructions that were built to describe all known metabolic reactions within the human body. Besides, a few tissue‐ and cell‐type‐specific models have been developed by incorporating tissue‐ or cell‐specific transcriptomics and proteomics data. These models can be used to predict possible ranges of metabolic fluxes for all enzymes in the network. Flux ranges in diseased and control (CTRL) models can be compared to discover functional changes in the metabolic network. These may be used as biomarkers or give insight into the biochemical origin of disease symptoms [11, 12, 13, 14, 15].

In the last decade, a paradigm shift occurred in the field of inborn errors of metabolism. Today, they are no longer viewed according to the ‘one gene, one disease’ paradigm as proposed more than 100 years ago, but recognised to be complex diseases [16]. However, only few studies using systems biology and multi‐omics approaches that are widely used for complex diseases have been published for inborn errors of metabolism [8, 9, 14, 17, 18, 19, 20, 21, 22].

In this study, we aim to investigate the metabolic phenotype of RD at the genome scale and to study the effect of phytanate on cell metabolism. Cultured fibroblasts contain most metabolic functions present in the human body, and biochemical and functional studies in cultured skin fibroblasts are important tools for the diagnosis of patients with a peroxisomal disorder [23]. Therefore, we reconstructed a fibroblast‐specific genome‐scale model based on fibroblast‐specific transcriptomics, metabolomics and proteomics data, and starting from the recently published Recon3D‐based model. We obtained these data from healthy CTRLs and RD patient fibroblasts incubated with phytol, a precursor of phytanate. Since flavoproteins play a crucial role in lipid metabolism, we integrated our recently curated set of FAD‐related reactions [20]. The resulting model reflects the *in vivo* situation in fibroblasts and demonstrates the physiological effects of a defective α‐oxidation. Ultimately, such insights in metabolic changes may provide leads for pathophysiology and therapy.

## Results

### Model curation and generating a fibroblast‐specific model

**Fig. 1:**
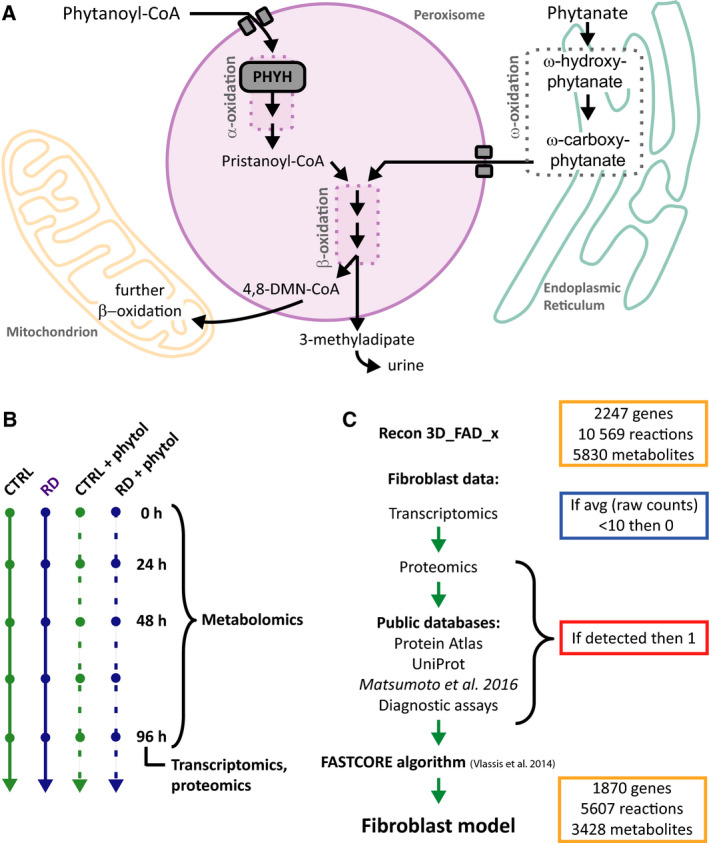
Developing a fibroblast‐specific model. (A) Schematic overview of relevant metabolic pathways for phytanate metabolism. (B) Schematic representation of the experimental set‐up. CTRL and RD fibroblasts were incubated with or without phytol, the precursor of phytanate, for the indicated time points. All cells were seeded and harvested under the same conditions. (C) Schematic overview of the steps to obtain a fibroblast‐specific model based on constraints of the Recon3D_FAD_x model.

For this study, we used an updated version of the Recon3D model in which flavoprotein‐related metabolism was curated [20]. This addition was essential for this study because many enzymes in fatty acid metabolism are flavoproteins, which carry FAD as a cofactor. Furthermore, a known alternative route for phytanate degradation, ω‐oxidation, was not accounted for in Recon3D. In this pathway, phytanate is first converted into ω‐hydroxyphytanate, followed by oxidation to the corresponding dicarboxylic acid (ω‐carboxyphytanate; see Fig. 1A). After activation to their CoA esters, dicarboxylic acids have been shown to enter the peroxisome by active transport via the ATP binding cassette transporter ABCD3 (also known as peroxisomal membrane protein PMP70) [24], and are then degraded via peroxisomal β‐oxidation [25]. It is assumed that ω‐carboxyphytanate follows the same pathway as an unbranched‐long‐chain dicarboxylic acid. The final product of phytanate breakdown via ω‐oxidation is 3‐MAA, which has been identified in urine from patients with RD [26].

To optimise the model, we added 25 reactions involved in the ω‐oxidation and the subsequent β‐oxidation of phytanate. Furthermore, 17 reactions involved in phytanate metabolism were deleted, because they were duplicates of other reactions in the model. Lastly, we examined the import/export reaction boundaries and blocked the flux of several drug metabolism pathways, such as those of statins, ibuprofen, paracetamol and antibiotics. These pathways were not relevant to this study but could play a role in the model outcome. All changes to the model are summarised in Table S1. The resulting curated model was called Recon3D_X_c and is available on GitHub (https://github.com/WegrzynAB/Papers).

**Fig. 2:**
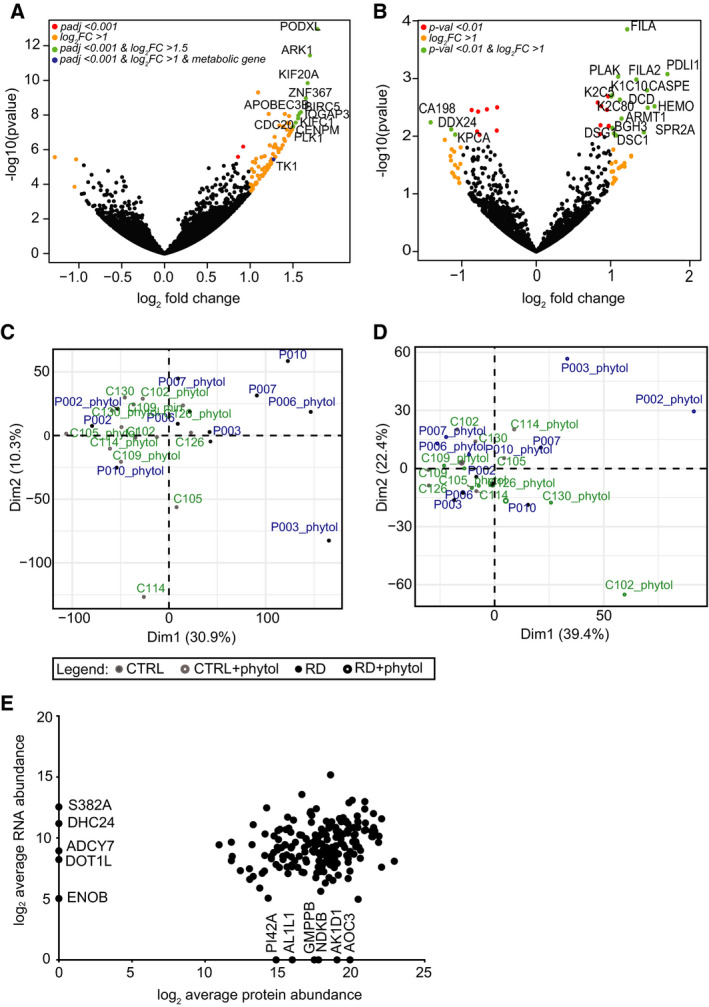
(A + B) Volcano plots depicting (A) the transcriptomics data and (B) proteomics data derived from fibroblasts incubated with phytol for 96 h. Genes and proteins, resp., with significant differences in expression between the diseases (RD) and CTRL groups are indicated with coloured dots. Gene names are shown for genes and proteins, resp., indicated with green dots. Blue dots represent metabolic genes, as included in the Recon3D model, that were expressed differentially at the significance level below 0.001, and their expression levels were changed by minimum onefold. (C + D) PCA for (C) transcriptomics data and (D) proteomics data. Raw expression values have been scaled and centred. (E) Correlation plot showing log_2_ average abundance of all proteins (*x*‐axis) and genes (*y*‐axis) that were included in the Recon3D model.

To create a fibroblast‐specific model, we generated a fibroblast dataset related to the metabolic genes included in the model. To this end, we cultured human primary CTRL fibroblasts (*n* = 6) and RD patient‐derived fibroblasts with a defect in α‐oxidation (*n* = 5) under standardised conditions, and harvested cells after 96 h to isolate RNA and protein. The cells were either incubated with phytol, a precursor of phytanate, or with the solvent ethanol (Fig. 1B). Our primary dataset consisted of the data obtained from transcriptomics (RNAseq) and proteomics (shotgun) measurements. In the principal component analysis (PCA), no separation was seen between the groups of fibroblasts (Fig. 2C,D), suggesting that overall the patient‐to‐patient variation was larger than the adaptation to the disease, at least in the fibroblasts. Differential analysis of the transcriptomics and proteomics data revealed only 12 differentially expressed genes and 18 proteins between the CTRL fibroblasts and fibroblasts defective in α‐oxidation (Fig. 2). All differentially expressed genes and 15 proteins were upregulated in the Refsum group relative to CTRLs, while no genes and only three proteins were downregulated. These upregulated genes and proteins were primarily involved in cell cycle CTRL and structure (Table S4). When we tested the correlation between protein and RNA levels in the subset of genes that were included in our database, six proteins that were detected in the shotgun proteomics were not present in the transcriptomics data, even though protein and RNA fractions were obtained from the same sample (Fig. 2C). To complement our own data, we included publicly available information of tissue‐specific gene and protein expression levels present in the Human Protein Atlas (Uhlen *et al*. [27], www.proteinatlas.org), published transcriptomics and proteomics data obtained from fibroblasts [28], OMIM information [29], fibroblast‐specific information published along with the Recon 2 model [8] and information on metabolic assays that are used for diagnostic approaches in fibroblasts (Table S2). To generate the fibroblast‐specific model, the activity of metabolic reactions was constrained in a two‐step manner (Fig. 1C). First, all genes involved in metabolic pathways that were not detected in our transcriptomics data with < 10 raw counts were initially marked as ‘inactive’. Secondly, all these genes were manually cross‐examined with our generated database to determine whether the gene was expressed in fibroblasts (either on RNA or on protein level). If expressed in skin fibroblasts, the gene was changed to ‘active’. Finally, the FASTCORE algorithm [30] was used to create a flux consistent fibroblast‐specific network. This procedure resulted in the final model, ‘fibroblast_CTRL’, which was used for further analysis.

### Model characterisation

First, we tested whether the fibroblast‐specific model showed physiological resemblance to fibroblasts *in vivo.* To this end, we used a set of metabolic tasks defined by Thiele *et al*. [8] and focused explicitly at the metabolic tasks, which are known to be crucial for fibroblast metabolism, that is the conversion of glutamine to α‐ketoglutarate [31]), or which are known to be absent in fibroblasts, that is bile acid metabolism [32]. The fibroblast‐specific model completed 208 out of all 419 generic tasks (Table S5), demonstrating that the fibroblast model adequately reflects general human metabolism. Additionally, specific reactions known to be present or absent in fibroblasts were also accurately predicted (Table 1), including diagnostically relevant genes (Table S5).

**Table 1:** Model performance in the metabolic tasks test. A subset of tasks relevant to fibroblast metabolism selected. For a full list of all tested tasks, see Table S5

Metabolic task	Reported in fibroblasts	Active in the model
Bile acid metabolism	NO	NO
Pyrimidine degradation	NO	NO
Glutamine to citrulline conversion	NO	NO
Melatonin synthesis	NO	NO
Urea cycle	NO	NO
Glutamine conversion to α‐ketoglutarate	YES	YES
ATP production via electron transport chain	YES	YES
Mitochondrial β‐oxidation	YES	YES
Peroxisomal β‐oxidation	YES	YES
Peroxisomal α‐oxidation	YES	YES
ω‐Oxidation of phytanate	YES	YES
All 419 generic metabolic tasks		208

Subsequently, we simulated the capacity of the fibroblast‐specific model to produce ATP from phytanate as the single‐carbon source under aerobic conditions in a minimal medium (consisting of only ions, oxygen, water and riboflavin). ATP utilisation is explicitly defined in the model and is corrected for ATP investments required for ATP synthesis, such as reactions involved in cofactor synthesis, metabolite transport and substrate activation. The ATP utilisation flux was used as an objective function of which the value was maximised in the steady‐state calculation. Since the flux through the ATP utilisation reaction equals that of ATP production after subtraction of ATP costs at steady state, it reflects the *net* ATP production from a single‐carbon source (in this case phytanate). In contrast to the initial Recon3D_FAD model, the curated model (Recon3D_FAD_X) and the fibroblast‐specific model (fibroblast_CTRL) showed a net ATP production flux of 68.5 and 61.65 mmol·gDW^−1^·h^−1^, respectively, at a forced phytanate uptake flux of 1 mmol·gDW^−1^·h^−1^.

**Fig. 3:**
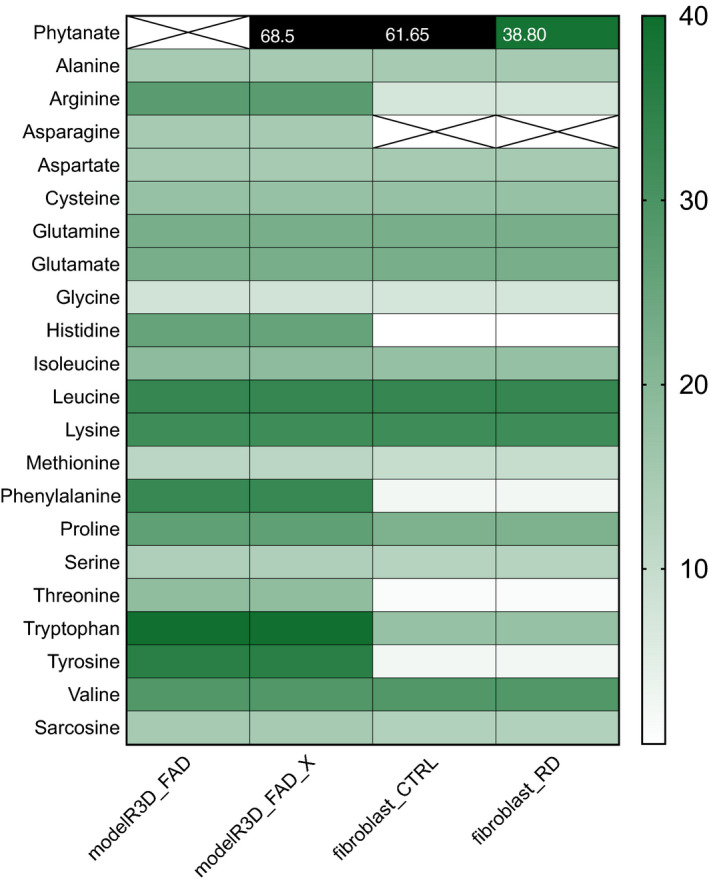
Model predictions of ATP yields from a single‐carbon source. Assessment of carbon source utilisation on minimal media based on the ATP production from single‐carbon source, including Recon3D_FAD, curated Recon3D_FAD for phytanate metabolism (Recon3D_FAD_x), the fibroblast‐specific model for CTRL (fibroblast_CTRL) and diseased conditions (fibroblast_RD). Green shades in the heatmaps reflect the relative net ATP production ranging from no (white) to high (dark green), and very high ATP production (black). Crossed‐out fields symbolise model inability to metabolise a carbon source (infeasible solution) on minimal media.

**Fig. 4:**
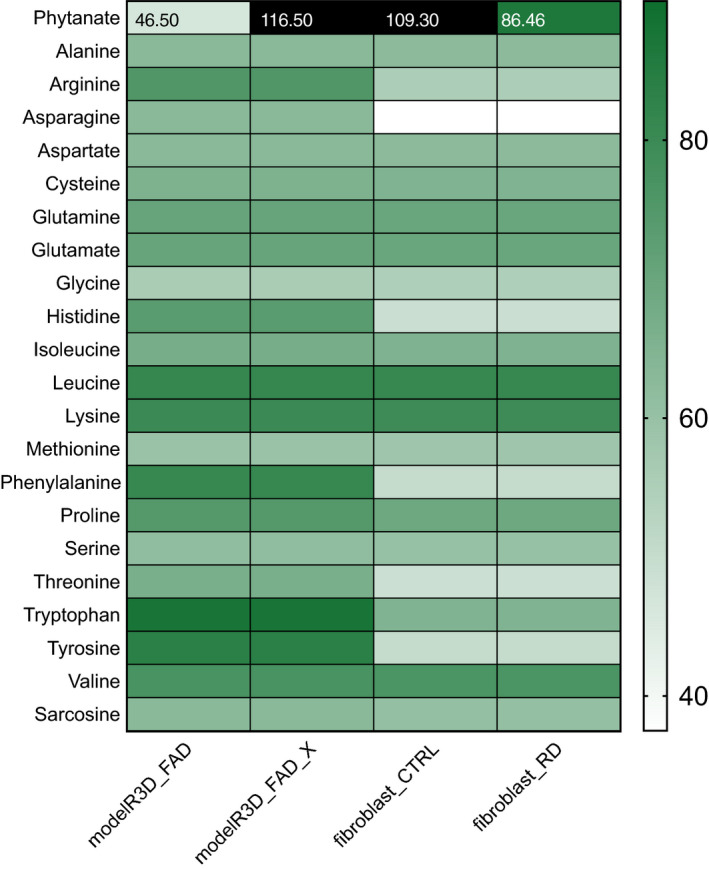
Model predictions of ATP yields from a single‐carbon source. Assessment of carbon source utilisation on minimal media supplemented with glutathione and pantothenic acid based on the ATP production from single‐carbon source, including Recon3D_FAD, curated Recon3D_FAD for phytanate metabolism (Recon3D_FAD_x), the fibroblast‐specific model for CTRL (fibroblast_CTRL) and diseased conditions (fibroblast_RD). Green shades in the heatmaps reflect the relative net ATP production ranging from no (white) to high (dark green), and very high ATP production (black).

Furthermore, we created a RD model (fibroblast_RD) by setting the flux through the phytanoyl‐CoA hydroxylase (PHYH, HGNC:8940) reaction to 0. The fibroblast_RD model was able to metabolise phytanoyl‐CoA in minimal medium conditions (Fig. 3, Table S3), albeit at a much lower flux than CTRL (38.8 mmol·gDW^−1^·h^−1^). These results implied that ω‐oxidation of phytanate and the subsequent β‐oxidation in the peroxisomes are less efficient in the ATP production and could require a richer growth media supplemented with glutathione (Fig. S2, uptake flux for glutathione was set at 1 mmol·gDW^−1^·h^−1^). Supplementation of glutathione to the minimum media allowed all studied models to break down phytanate, albeit with very strong differences in the total ATP yields. The net ATP production flux of 46.50 and 86.46 mmol·g·DW^−1^·h^−1^ was shown for the initial Recon3D_FAD model and the fibroblast_RD model, respectively, while much higher net ATP production flux of 116.5 and 109.3 mmol·gDW^−1^·h^−1^ was seen for the Recon3D_FAD_X and the fibroblast_CTRL models (Fig. 4). Similarly, we analysed the amino acid catabolism in the models. All amino acids could be catabolised to yield ATP in the Recon3D_FAD_X model. However, the fibroblast‐specific models were unable to metabolise asparagine, histidine and threonine, as well as nearly no ATP yield from phenylalanine and tyrosine. Furthermore, net ATP production from tryptophan was lower in fibroblast‐specific model compared with the generic model (Fig. 3). In the minimum media supplemented with glutathione and pantothenic acid, all amino acids were broken down; however, asparagine, histidine, phenylalanine, threonine and tyrosine were showing a strong decrease in the ATP yield in the fibroblast models compared with the generic models (Fig. 4).

**Fig. 5:**
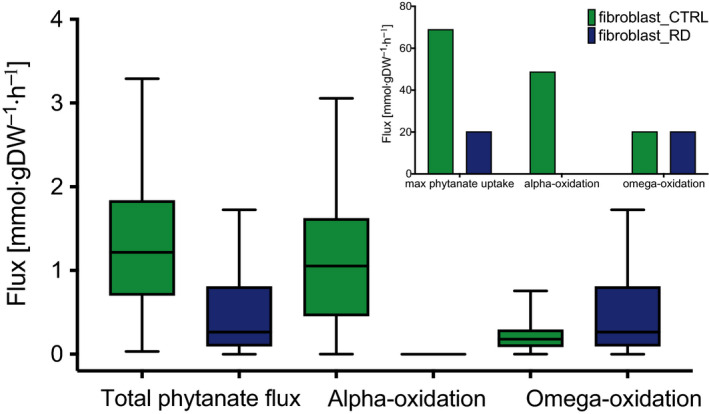
Simulation of phytanate metabolism. Missing reactions for phytanate metabolism, including α‐ and ω‐oxidation, were added to the Recon3D_FAD model. The curated, fibroblast‐specific model shows differences in metabolic fluxes of phytanate through the available pathways under normal (CTRL, green) and diseased conditions (RD, blue). Insert shows maximised fluxes, including blocked α‐oxidation in PHYH conditions. Predictions are shown as box‐and‐whisker (the horizontal line at median, and whiskers at min and max values, *n* = 10 000) plots (main figure), or bar plots (insert).

To investigate the effect of a defective α‐oxidation on the flux distribution in the curated, fibroblast‐specific model, we used the fibroblast_RD model to sample the steady‐state solution space using the ACHR algorithm [33]. Since genome‐scale models typically have multiple steady‐state solutions, in this procedure, the solution space reflects the flux ranges found for each reaction when sampling many steady‐state solutions (see Materials and methods for details). To be able to compare the results of this analysis with the data from the *in vitro* fibroblast studies, rich media were used. As expected, the total flux of phytanate uptake into the cell was decreased in the fibroblast_RD model when compared to the fibroblast_CTRL model. Because of the simulated deletion of the PHYH gene, α‐oxidation was abolished entirely in the fibroblast_RD model, whereas it was active in the fibroblast_CTRL model (Fig. 5). Pathways involved in ω‐oxidation, however, were active in both models (Fig. 5). Interestingly, both pathway fluxes were significantly smaller than their maximum rates as obtained from the simulation wherein the maximum flux of α‐ or ω‐oxidation pathways was used as objective functions (Fig. 5, insert).

### Metabolic characterisation of fibroblasts cultured *in vitro*

**Fig. 6:**
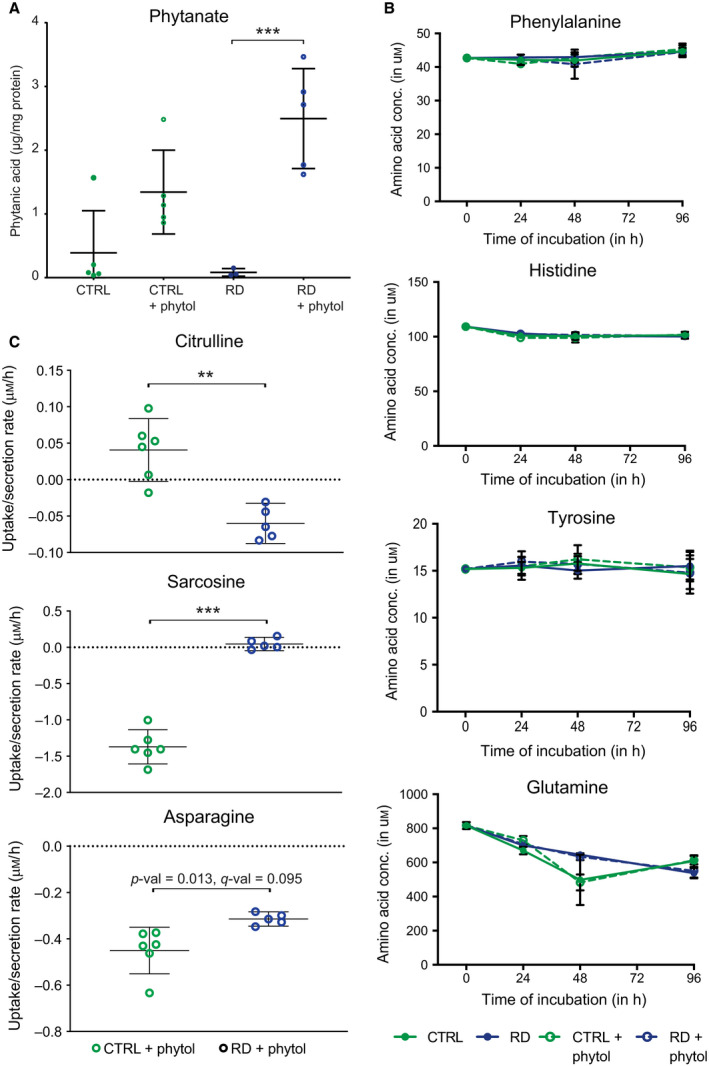
Metabolic characterisation of fibroblasts cultured *in vitro*. Model validation using experimental data of (A) phytanate, and (B + C) amino acid measurements. (A) Phytanate concentrations were determined in pellets from cultured cells after incubation for 96 h. Phytanate levels are increased in cells incubated with phytol. Per condition, mean per group and 95% confidence interval per group are indicated. Significant differences between the groups were determined by one‐way ANOVA (***P‐value < 0.001, *n* CTRL = 6, *n* RD = 5). (B) Significantly changed uptake and secretion rates of amino acids between healthy (*n* = 6) and RD (*n* = 5) fibroblasts exposed to phytol for 96 h (shown as mean and 95% confidence interval). Amino acid concentrations were determined in the medium of the cells 96‐h incubation with phytol. Rates were calculated based on the fresh medium measurements. Significant differences between the groups were determined using a *t*‐test with a two‐stage linear step‐up procedure of Benjamini, Krieger and Yekutieli, with Q = 1%, to correct for the multiple testing (**q‐value < 0.01, ***q‐value < 0.001). Rates of uptake and secretion of other amino acids are shown in Fig. S1. C) Amino acid concentrations were determined in the medium of the healthy (*n* = 6) and RD (*n* = 5) fibroblast cells after incubation at indicated time points (shown as mean ± SD). Results for other amino acids are shown in Fig. S2D.

To qualitatively validate our model predictions, we obtained fibroblast‐specific metabolomics data. Similar to the transcriptomics and proteomics experiments, we cultured human primary CTRL fibroblasts (*n* = 6) and RD patient‐derived fibroblasts (*n* = 5) under standardised conditions, and collected cell culture medium and cells every 24 h for four consecutive days. The cells were incubated with phytol or with the solvent ethanol (Fig. 1B). First, we measured the levels of total phytanate in cells incubated with or without phytol for 96 h. The addition of phytol resulted in increased levels of phytanate when compared to untreated cells. This was expected, as phytol is converted to phytanate once taken up into the cell [34]. In addition, phytanate levels were increased in fibroblasts with a defect in α‐oxidation when compared to CTRL fibroblasts when phytol was added to the medium (Fig. 6A), reflecting impaired oxidation of phytanate.

Furthermore, we measured amino acid profiles in the cell culture medium. We observed no significant changes between the CTRL and RD groups (Fig. 6B and Fig. S2A) at measured time points. However, a few changes were seen in the rates of uptake or secretion of amino acids (Fig. 6C and Fig. S1). Notably, citrulline and sarcosine have shown to change the directionality in the two groups. While citrulline is secreted, and sarcosine is consumed in the healthy fibroblasts exposed to phytol for 96 h, this situation is reversed in RD fibroblasts. Furthermore, uptake of asparagine is decreased in the RD fibroblasts compared with the healthy ones (Fig. 6C). Other amino acids show some minor differences in their uptake or secretion rates; however, those are not significant (Fig. S1).

Finally, glucose levels (Fig. 4B), cellular protein levels (Fig. 4C) and cell content (Fig. 4D) were similar between the CTRL fibroblasts and the RD fibroblasts with a defect in α‐oxidation after 96 h of cell culture.

### Predicting physiological effects of defective α‐oxidation

**Fig. 7:**
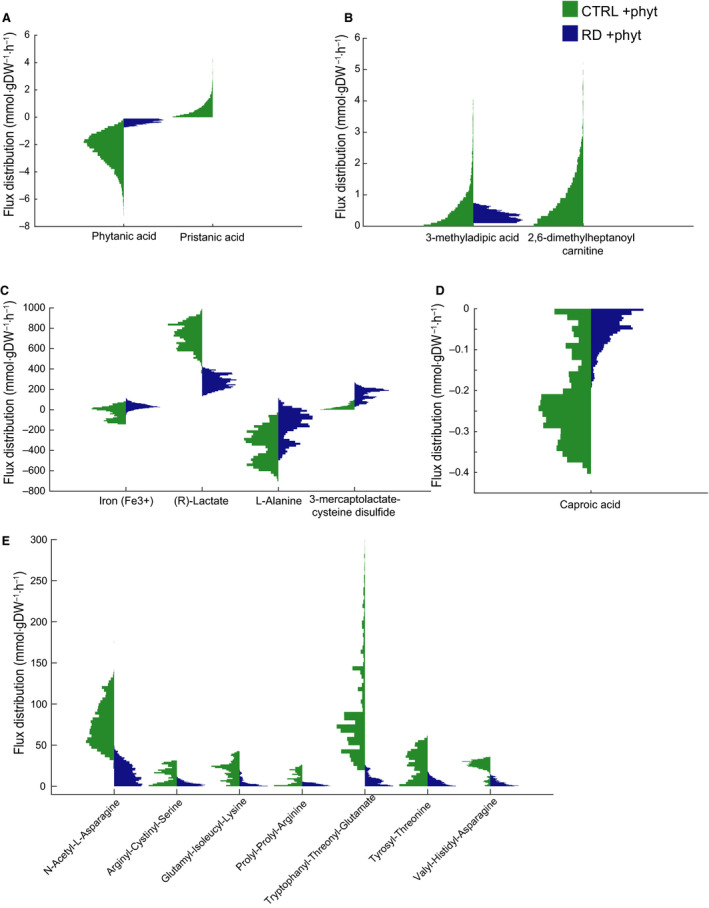
Changes at the level of secretion and uptake reactions between healthy and Refsum models forced to take up phytanate. (A–E) Secretion/uptake fluxes distributions of metabolites with the most significant differences between CTRL (CTRL + phyt, green; *n* = 10 000) and RD (RD + phyt, blue, *n* = 10 000) models forced to take up phytanate selected based on the log_2_(FC) > 1.3 and FDR < 0.05. Statistical differences were analysed using the Wilcoxon rank sum test; FDR values were calculated using the Bonferroni–Holm correction.

To investigate other flux changes in the fibroblast_RD model when compared to the fibroblast_CTRL model, we explored the steady‐state flux distribution obtained by the sampling of the solution space in the model. We studied changes in the flux ranges of the exchange reactions between CTRL and disease models after forcing a minimum uptake of phytanate 0.1 mmol·gDW^−1^·h^−1^) in the models. Shlomi *et al*. [19] proposed that if the secretion flux through the exchange reaction is high, it may lead to a high metabolite concentration outside of the cell. In contrast, if uptake is more prevalent, then the extracellular concentration is expected to be lower under the studied conditions. Exchange reactions in the model define the model boundaries. They allow some metabolites to be imported in or secreted from the cell, enabling the model to reach a steady state. First, we compared the model predictions for the exchange of amino acids with the data obtained *in vitro*. Our model predicted the directionality of amino acid exchanges with 73% accuracy, in line with the previously published accuracy scores [8, 20] (Table S6). Secondly, we investigated the response of the two models to phytanate. They responded differently to the forced phytanate uptake flux (Fig. 7). The mean value of the phytanate flux was reduced by 85% in the fibroblast_RD model when compared to the fibroblast_CTRL model, and secretion of pristanic acid was absent in the RD model (Fig. 7A). The export reaction of 3‐MAA, which is the end product of subsequent ω‐ and β‐oxidation of phytanate (Figs 1A and 7B), did not show a significant change in its mean flux, while 2,6‐dimethylheptanoyl carnitine, one of the end products of canonical degradation pathway of phytanate, showed 100% decrease of the flux rate in RD. Besides these known metabolites associated with a defect in α‐oxidation, we identified 49 other boundary metabolites that were significantly changed (FDR < 0.05 and log_2_FC > 1.3) between the fibroblast_RD and the fibroblast_CTRL models (Table S6). Of these, 24 flux changes were predicted to lead to higher extracellular concentrations in the absence of PHYH activity, including l‐alanine and 3‐mercaptolactate‐cysteine disulphide (Fig. 7C), caproic acid (Fig. 7D), 2‐hydroxybutyrate, malonylcarnitine and several di‐ and tripeptides (Table S7). On the other hand, 27 distribution flux changes were predicted to result in reduced extracellular concentrations in RD fibroblasts, such as lactate (Fig. 7C), *N*‐acetyl‐asparagine, l‐citrulline (Table S7) and several di‐ and tripeptides (Fig. 7E and Table S7). These changes depend either on the lower/higher uptake rate or on a lower/higher secretion rate (Fig. 7C–E). Interestingly, the rate of secretion of citrulline in our *in vitro* study showed a significant decrease (Fig. 6C) confirming one of our model predictions.

## Discussion

In this study, we present a fibroblast‐specific metabolic model for RD. Using transcriptomics and proteomics data, we developed a cell‐specific metabolic network based on Recon3D_FAD [20]. Cell‐type‐specific metabolic models have been reported earlier [13, 14, 17, 21, 22, 35], and are essential tools to study specific research questions. We studied the effect of phytanate loading on the metabolic fluxes in a fibroblast‐specific model for RD, which is characterised by a defect in α‐oxidation. Phytanate is a natural ligand of peroxisome proliferator receptor α (PPARα) [3, 4]. Furthermore, elevated levels of phytanate have been reported to induce lipotoxicity in the brain [36]. Many of these findings, however, derive from *in vitro* experiments. The consequences of phytanate accumulation have also been studied in a mouse model of RD, which resembles the clinical symptoms of patients [3, 37]. Notably, these mice showed no disease phenotype when fed a regular diet, but only developed the phenotype resembling RD when challenged with a phytol‐enriched diet [37]. Studies in humans, however, are scarce due to limited options for invasive studies. Computational modelling of human cells or tissues is meant to fill this gap partly. In our study, we curated the existing genome‐scale model by including pathway information for ω‐oxidation and following β‐oxidation of phytanate and constrained the model to obtain a fibroblast‐specific model based on generated data and existing databases. The reconstruction of metabolic networks is an iterative process, and updates will assure better accuracy and prediction of the human metabolic model [38]. Our model predicted amino acid fluxes with 73% accuracy in line with the previously reported values [8, 20]. To reach higher accuracy, further curation of the network might be required. Since amino acids are the main building blocks of the cell, a cell‐type‐specific protein composition incorporated in the biomass function of the model might yield more accurate results.

Using the curated model, we aimed to get an insight into metabolic changes that may provide leads for pathophysiology and biomarkers. Genome‐scale metabolic models have been described to be useful tools for these aims [8, 9, 10, 14, 19, 20, 21, 22, 35]. In our fibroblast‐specific model resembling RD, the flux of phytanate uptake was significantly reduced, reflecting the accumulation of phytanate in the body, a known biomarker for RD [3]. On the other hand, the average 3‐MAA secretion rate was not changed between the models. Our results show that it is more desirable for metabolism to lower the phytanate uptake rather than increase the ω‐oxidation. However, an average sampled flux of 3‐MAA secretion was 60 times as low as its maximum theoretical yield (Fig. 5B), showing that the ω‐oxidation pathway can be upregulated further. Notably, it has been described that ω‐oxidation was indeed upregulated in patients with peroxisomal disorders [4, 39].

Besides the changes in the known biomarkers, the model predicted aberrant flux distributions, leading to accumulation or reduction of extracellular metabolites in the Refsum fibroblast model when compared to the healthy model. Interestingly, di‐ and tripeptides were predicted to be changing significantly between the patient and healthy models (Fig. 7F). Biologically active peptides [40] have been found to play important roles in the metabolic functions, including intercellular signal transmission [41] and neuron signal transmission [42, 43]. Furthermore, specific peptides are involved in the processes that lead to disease development, and their presence could indicate specific diseases, that is serve as disease biomarkers [44, 45, 46, 47]. However, the power of the prediction and the value of these metabolic changes in relation to the pathogenesis of phytanate in patients with RD require further analysis. If validated, our predictions could lead to potential therapeutic strategies to intervene with the accumulation of phytanate in these patients. The upregulation of ω‐oxidation as an escape route for the breakdown of phytanate, and also very‐long‐chain fatty acids, has been studied *in vitro* for diseases such as RD and X‐linked adrenoleukodystrophy [3, 39]. The activation of the cytochrome P450 family (CYP) 4A enzymes, which are known to induce ω‐oxidation, has indeed been an attractive target for therapeutic interventions. However, until now, studies using compounds or drugs to upregulate ω‐oxidation via CYP4A have not been performed successfully [48]. Our model predicts (Figs 3 and 4) that increase in the glutathione levels could not only protect the cells from the oxidative stress postulated to play a role in RD [49] but potentially also support the phytanate breakdown via the ω‐oxidation pathway. However, the clinical value of our predictions remains to be evaluated. Fortunately, as mentioned before, a mouse model of RD exists in which a systemic whole‐body effect of phytanate accumulation has been studied [37]. Since the expression of ω‐hydroxylases from the CYP4 family is similar in mice and humans, studies using mice fed with a glutathione‐enriched diet could be performed to determine the rate of ω‐oxidation of phytanate. To investigate the clinical potential of our findings, these diet studies could be complemented with the application of previously proposed CYP4 inducers, that is fibrates and statins [4].

## Materials and methods

### Cell culture

For this study, we used anonymised primary skin fibroblasts from patients that had been sent previously to our laboratory for diagnostic evaluation and that were diagnosed with RD. All cell lines were anonymised. Fibroblasts were cultured in 75‐cm^2^ flasks for transcriptomics and proteomics analysis, and in 25‐cm^2^ flasks for metabolomics experiments. Cells were cultured in Ham's F‐10 medium with l‐glutamine, supplemented with 10% FBS (Invitrogen, Carlsbad, CA, USA), 25 mm HEPES, 100 U·mL^−1^ penicillin and 100 µg·mL^−1^ streptomycin, and 250 µg·mL^−1^ amphotericin in a humidified atmosphere of 5% CO_2_ at 37 °C. Cells were seeded on the same day and incubated for the indicated time points (Fig. 1B). To standardise tissue culture conditions, cells were grown to 100% confluence in the flask, which was achieved after 96 h of incubation. Cells were incubated with 25 μm phytol, dissolved in ethanol, or ethanol as the vehicle. Cells were harvested by trypsinisation (0.5% trypsin/EDTA; Invitrogen) and washed once with phosphate‐buffered saline and twice with 0.9% NaCl, followed by centrifugation at 4 °C (16 100 ***g*** for 5 min) to obtain cell pellets. For metabolomics experiments, the cell culture medium was collected before harvesting. Cell pellets and medium samples were stored at −80 °C until analysis.

### RNA and protein isolation for RNAseq and Shotgun proteomics measurements

RNA and protein were isolated from the cell pellets from the T75 cultures using TRIzol^TM^ Reagent (Thermo Fisher Scientific, Waltham, MA, USA) using supplier protocol for RNA and protein extraction. RNA pellets were dissolved in 50 μL of RNase‐free water, and RNA concentrations were measured using NanoDrop™ 2000 Spectrophotometer (Thermo Fisher Scientific). Protein pellets were dissolved in 200 μL 5% SDS solution, and protein concentrations were determined using Pierce™ BCA Protein Assay Kit (Thermo Fisher Scientific).

### RNAseq

#### Sample preparation and sequencing

First quality check of and RNA quantification of the samples were performed by capillary electrophoresis using the LabChip GX (PerkinElmer, Waltham, MA, USA). Nondegraded RNA samples were selected for subsequent sequencing analysis. Sequence libraries were generated using the NEXTflex Rapid Illumina Directional RNA‐Seq Library Prep Kit (Bioo Scientific, Austin, TX, USA) using the Sciclone NGS Liquid Handler (PerkinElmer). The obtained cDNA fragment libraries were sequenced on an Illumina Nextseq500 (Illumina, San Diego, CA, USA) using default parameters (single read 1 × 75 bp) in pools of multiple samples, producing on average 4 million reads per sample.

#### Gene expression quantification

The trimmed fastQ files were aligned to build human_g1k_v37 Ensemble [50] release 75 reference genome using hisat/0.1.5‐beta‐goolf‐1.7.20 [51] with default settings. Before gene quantification, SAMtools/1.2‐goolf‐1.7.20 [52] was used to sort the aligned reads. The gene‐level quantification was performed by HTSeq‐count: HTSeq/0.6.1p1 [53] using ‐‐mode=union, with Ensembl release 75 [50], was used as a gene annotation database.

#### Calculate QC metrics on raw and aligned data

Quality CTRL (QC) metrics are calculated for the raw sequencing data. This is done using the tool FastQC (FastQC/0.11.3‐Java‐1.7.0_80) [54]. QC metrics are calculated for the aligned reads using Picard‐tools (picard/1.130‐Java‐1.7.0_80) [55] CollectRnaSeqMetrics, MarkDuplicates, CollectInsertSize‐ Metrics and SAMtools/1.2‐goolf‐1.7.20 flagstat.

### Shotgun proteomics

#### In‐gel digestion and strong cation‐exchange fractionation

Protein samples were mixed with LDS loading buffer (NuPAGE) at a concentration of 3.4 µg total protein. The sample was run briefly into a precast 4–12% Bis‐Tris gels (Novex, Carlsbad, CA, USA, ran for maximally 5 min at 100 V). The gel was stained with Biosafe Coomassie G‐250 stain (Bio‐Rad, Redmond, WA, USA), and after destaining with milliQ‐H_2_O (Merck, Burlington, MA, USA), the band containing all proteins was excised from the gel. The gel band was sliced into small pieces, and washed subsequently with 30% and 50% v/v acetonitrile in 100 mm ammonium bicarbonate (dissolved in milliQ‐H_2_O), each incubated at RT for 30 min while mixing (500 r.p.m.), and lastly with 100% acetonitrile for 5 min, before drying the gel pieces in an oven at 37 °C. The proteins were reduced with 20 μL 10 mm dithiothreitol (in 100 mm ammonium bicarbonate dissolved in milliQ‐H_2_O, 30 min, 55 °C) and alkylated with 20 μL 55 mm iodoacetamide (in 100 mm ammonium bicarbonate dissolved in milliQ‐H_2_O, 30 min, in the dark at RT). The gel pieces were washed with 50% v/v acetonitrile in 100 mm ammonium bicarbonate (dissolved in milliQ‐H_2_O) for 30 min while mixing (500 r.p.m.) and dried in an oven at 37 °C before overnight digestion with 20 μL trypsin (1 : 100 g·g^−1^, sequencing grade modified trypsin V5111; Promega, Madison, WI, USA) at 37 °C. The next day, the residual liquid was collected before elution of the proteins from the gel pieces with 20 µL 75% v/v acetonitrile plus 5% v/v formic acid (incubation 20 min at RT, mixing 500 r.p.m.). The elution fraction was combined with the residual liquid and was dried under vacuum and resuspended in 30 μL of 20% v/v acetonitrile plus 0.4% v/v formic acid (dissolved in milliQ‐H_2_O) for strong cation‐exchange (SCX) fractionation. Samples were loaded onto an SCX StageTips (20 μL tip StageTip; Thermo Scientific) according to the manufacturer's instructions, except that the elution solvent (500 mm ammonium acetate in 20% v/v acetonitrile, dissolved in milliQ‐H_2_O) plus 0.4% v/v formic acid was used instead of the 1 m NaCl solution in this protocol during initialisation. After loading and washing of the peptides according to the protocol, the peptides were eluted in three separate fractions by stepwise elutions (30 μL each) of 25, 150 and 500 mm ammonium acetate in 20% v/v acetonitrile (dissolved in milliQ‐H_2_O). The collected flow‐through was polled with the last elution fraction. The elution fractions were dried under vacuum and resuspended in 8 μL 0.1% v/v formic acid (dissolved in milliQ‐H_2_O).

#### LC‐MS analysis

Discovery mass spectrometric analyses were performed on a quadrupole–Orbitrap mass spectrometer equipped with a nano‐electrospray ion source (Orbitrap Q Exactive Plus; Thermo Scientific). Chromatographic separation of the peptides was performed by liquid chromatography (LC) on a nano‐HPLC system (Ultimate 3000; Dionex, Sunnyvale, CA, USA) using a nano‐LC column (Acclaim PepMapC100 C18, 75 µm × 50 cm, 2 µm, 100 Å; Dionex, buffer A: 0.1% v/v formic acid, dissolved in milliQ‐H_2_O, buffer B: 0.1% v/v formic acid, dissolved in acetonitrile). In general, 6 µL was injected using the µL‐pickup method with buffer A as a transport liquid from a cooled autosampler (5 °C) and loaded onto a trap column (µPrecolumn Cartridge, Acclaim PepMap100 C18, 5 µm, 100 Å, 300 µm × 5 mm; Dionex). Peptides were separated on the nano‐LC column using a linear gradient from 2% to 40% buffer B in 117 min at a flow rate of 200 nL·min^−1^. The mass spectrometer was operated in positive ion mode and data‐dependent acquisition mode using a top‐10 method. MS spectra were acquired at a resolution of 70 000 at *m*/*z* 200 over a scan range of 300–1650 *m*/*z* with an AGC target of 3e^6^ ions and a maximum injection time of 50 ms. Peptide fragmentation was performed with higher energy collision dissociation (HCD) using normalised collision energy of 27. The intensity threshold for ion selection was set at 2.0 e^4^ with a charge exclusion of ≤ 1 and ≥ 7. The MS/MS spectra were acquired at a resolution of 17 500 at *m*/*z* 200, an AGC target of 1e^5^ ions and a maximum injection time of 50 ms, and the isolation window was set to 1.6 *m*/*z*.

#### LC‐MS data analysis

Liquid chromatography‐MS raw data were processed with maxquant (version 1.5.2.8) [56]. Peptide and protein identification was carried out with Andromeda against a human SwissProt database (www.uniprot.org, downloaded 10 November 2016, 20 161 entries) and a contaminant database (298 entries). The searches were performed using the following parameters: precursor mass tolerance was set to 10 p.p.m., and fragment mass tolerance was set to 20 p.p.m. For peptide identification, two miss cleavages were allowed, a carbamidomethylation on cysteine residues as a static modification and oxidation of methionine residues as a variable modification. Peptides and proteins were identified with an FDR of 1%. For protein identification, at least one unique peptide had to be detected, and the match between run option was enabled. Proteins were quantified with the MaxLFQ algorithm [57] considering unique peptides and a minimum ratio count of one. Results were exported as tab‐separated *.txt for further data analysis.

### Principal component analysis and differential analysis of transcriptomics and proteomics

Principal component analysis was performed using prcomp function with raw data being first normalised (scaled and centred). Differential gene/protein expression analysis based on the negative binomial distribution was performed using DESeq2 [58]. Genes for which summed across all samples raw counts were higher than 20 were analysed. Protein intensities were transformed to integers and analysed similar to the transcriptomics data.

### Cell growth

Fibroblasts were seeded in 96‐well plate with a density of 2000 cells per well and cultured in 200 μL of medium for 7 days. xCELLigence system (ACEA Biosciences, Inc., San Diego, CA, USA) was used to monitor cell attachment and growth in real time [59]. Areas under the curve were calculated using prism7 (GraphPad Software, San Diego, CA, USA).

### Metabolomics

#### Determination of protein concentration in cell pellets

Cell pellets were sonicated in 250 μL of water. Protein concentration was determined using the Pierce™ BCA Protein Assay Kit (Thermo Fisher Scientific).

#### Amino acid profile

To analyse the amino acid profile of medium from cell cultures, 100 μL of the medium sample was mixed with 100 μL of internal standard (12 mg of norleucine mixed with 15 g sulphosalicylic acid in 250 mL of water). The analysis was performed according to the method of Moore, Spackman and Stein [60] on a Biochrom 30™ Amino acid Analyser (Biochrom.co.uk). Acquisition and data handling were done with Thermo Scientific™ Chromeleon™ 7.2 Chromatography Data System software (Thermo Fisher Scientific).

#### Sugar measurements

To analyse sugar profiles, 250 μL of the medium sample or 100 μL of a standard mix (50 mg of d‐(+)‐glucose in 50 mL of water) was mixed with 100 μL of internal standard (50 mg phenyl‐b‐d‐glucopyranoside in 50 mL of water mixed with 1 mL of chloroform). Glucose analysis was performed as described by Jansen *et al*. [61] on a Trace GCMS (Thermo Fisher Scientific). Acquisition and integrations were done with Xcalibur™ software (Thermo Fisher Scientific).

#### Phytanate measurement

Phytanate levels were measured as described previously [62].

### Model curation

Our model is based on a previously published FAD‐curated version of Recon3D [20]. Current representation of phytanate metabolism was analysed and compared with current knowledge [4, 63]. Missing reactions in omega‐oxidation of phytanate and follow‐up peroxisomal beta‐oxidation of its products were added to the reconstruction. Additionally, invalid or duplicated reactions (created by merge of different metabolic reconstructions to create Recon 2 model [8]) were removed. The curated model was saved as Recon3D_FAD_X. For detailed information on all the changes to the model, see Table S1 [fix, del].

### Model constraints

We examined all exchange/demand reactions to determine the model constraints. Since drug metabolism introduced by Sahoo *et al*. [64] is out of the scope of our research, we decided to block the import/export reactions for drugs and their metabolites. Additionally, we identified redundant demand and sink reactions that duplicate some exchange/demand reactions or allow sink reaction for a metabolite whose metabolism has been fully reconstructed and does not create a dead‐end pathway. Last, we closed all import reactions besides those that transported compounds present in the culture media, water and oxygen. All the changes can be examined in Table S1 (constraints).

Additionally, ‘biomass_reaction’ minimum flux was set to 0.1 mmol·gDW^−1^·h^−1^, to mimic the essential cell maintenance (protein synthesis, DNA and RNA synthesis etc.), unless stated otherwise, as in Ref. [65]. Other constraints used only in specific simulations are indicated where applicable.

### Fibroblast‐specific gene database

A database containing information about the expression levels of metabolic genes (genes present in the metabolic reconstruction Recon3D_FAD) and proteins in human fibroblasts was first generated based on the results from our transcriptomics and proteomics experiments. Additionally, we added information present in the Human Protein Atlas [27, 66], OMIM [29] fibroblast‐specific information published along with the first Recon 2 model [8] and UniProt [67] databases. Experimental data from human fibroblast gene expression levels by Matsumoto *et al*. [28] were also included. Usage of fibroblasts in diagnostics of specific gene defects was also examined. In the end, a binary decision was made about fibroblast‐specific genes – 1 if there was evidence for a gene/protein to be present in human fibroblasts, and 0 for genes classified as inactive in fibroblasts. Database, including the final decision, is available as Table S2.

### Fibroblast‐specific model generation

A list of reactions depending on the genes marked as active was used as a core set for the FASTCORE algorithm [30] implemented in The COBRA Toolbox v3.0 [68]. Next, reactions dependent on the inactive genes were removed, and the fastcc algorithm [30, 68] was used to generate a flux, consistent fibroblast‐specific model. The final model, named ‘fibroblast_CTRL’, is available in our GitHub folder.

### Model analysis

#### Refsum simulations

Phytanoyl‐CoA hydroxylase deficiency (RD) was simulated as a single gene deletion (PHYH, HGNC:8940). Additionally, ω‐oxidation (‘CYP450phyt’ reaction) and α‐oxidation (‘PHYHx’ reaction) pathway maximum rates were constrained to 20.2176 and 48.7656 mmol·gDW^−1^·h^−1^, respectively, to reflect those described in the literature [69, 70]. Lastly, the ‘EX_phyt(e)’ reaction upper boundary was set to −0.1 mmol·gDW^−1^·h^−1^ to force the model to utilise phytanate at a minimum rate of 0.1 mmol·gDW^−1^·h^−1^ for the simulations resembling fibroblasts with phytol added to the medium.

To sample the solution space of generated models, ACHR algorithm [33] implemented in the COBRA Toolbox 3.0 [68] was used. Randomly selected 10 000 sampled points were saved with from the total of 50 000 sampled points with a 500 step size.

#### Calculation of maximum ATP yield per carbon source

To calculate the maximum ATP yield per carbon source, we adapted the method developed by Swainston *et al*. [38]. Shortly, all uptake rates of nutrients were set to 0, except for a set of reactions defined collectively as a minimal medium (Ca^2+^, Cl^−^, Fe^2+^, Fe^3+^, H^+^, H_2_O, K^+^, Na^+^, ${\text{NH}}_{4}{\text{SO}}_{4}^{2-}$, Pi and riboflavin) for which the uptake/export fluxes rates were set to −1000 and 1000 mmol·gDW^−1^·h^−1^, respectively. For each of the specified carbon sources, the uptake flux was set to −1 mmol·gDW^−1^·h^−1^ forcing the model to consume it at a fixed rate. The demand reaction for ATP, ‘DM_atp_c_’ was used as an objective function flux, which should be maximised in the optimisation process. The oxygen intake flux was set to ‘EX_o2(e)’ −1000 mmol·gDW^−1^·h^−1^ to maintain aerobic conditions. If the model was unable to break down specified carbon source to ATP, the steady‐state flux could not be reached (infeasible solution).

#### Statistical analysis of model predictions

Flux distribution of each exchange reaction was compared between the CTRL and RD to find the most changed metabolite fluxes. To this end, we tested normality and variance of the distributions using single‐sample Kolmogorov–Smirnov goodness‐of‐fit hypothesis test and two‐sample *F*‐test for equal variances, respectively. Depending on the outcome, Student's *t*‐test (for normally distributed samples with equal or unequal variance) or Wilcoxon rank sum test (for non‐normally distributed samples with unequal variance) was used to determine whether the differences between the CTRL and RD models were significant. The Bonferroni–Holm correction for multiple comparisons was used to calculate the adjusted *P*‐values (FDR). Significance thresholds were set at FDR < 0.05 and log_2_(FC) > 1.3.

### Software

Model curation and all simulations were carried out with MATLAB R2019a (MathWorks, Inc., Natick, MA, USA) using the Gurobi 8.1 (Gurobi Optimization, Inc., Houston TX, USA) linear programming solver and the COBRA 3.0 toolbox [68].

## Conflicts of interest

The authors declare no conflict of interest.

## Author contributions

ABW, KH, RW, HW and BMB conceived the idea. ABW and KH designed the experiments. KH, AEML and ABW performed cell experiments. KH performed the phytanate measurement experiment. AG performed the metabolomics experiment. MK and JCW performed the proteomics experiments. AMD and ABW performed cell growth experiments. ABW performed computational modelling. ABW and KH analysed the data. ABW and KH wrote the first draft of the manuscript. All authors participated in the reviewing and editing of the subsequent drafts.

## Supporting information


**Table S1.** Manual curation of the model including added and curated reactions [Fixes], deleted reactions [ Recon3D_del], added metabolites [Added_mets], and information about the media constraints [HAM'sF10].
**Table S2.** Fibroblast specific genes.
**Table S3.** Detailed information about the ATP yields for Figs 3 and 4.
**Table S4.** Genes and proteins significantly changed between RD and CTRL fibroblasts.
**Table S5.** Detailed results of the metabolic tasks analysis in the fibroblast model.**Table S6.** Comparison between the exchanged amino acids in model prediction and in the *in vitro* experiments (CTRL + phyt group). For a full list of predicted uptake and secretion rates of metabolites see Table S7.
**Table S7.** Detailed comparison at the level of secretion and uptake reactions between healthy and Refsum models forced to take up phytanate.
**Fig. S1.** Additional data on amino acid uptake and secretion rates in the fibroblast CTRL (*n* = 6; green) and RD (*n* = 5; blue) cultures exposed to phytol for 96 h (shown as mean ± SD). Rates were calculated based on the fresh medium measurements.
**Fig. S2.** Additional experimental data of (A) amino acids and (B) glucose determinations in the medium of the fibroblast CTRL (*n* = 6) and RD (*n* = 5) cells after incubation at indicated time points (shown as mean ± SD). For details, see Fig. 6C. (C) Protein concentrations of cell pellets after incubation at indicated time points. (D) Growth curves of attached cells for the indicated time points (left panel), and statistical analysis of the total area under the curve per cell line after 7 days of incubation (right panel). Data are shown as bar plots.

## Data Availability

The mass spectrometry proteomics data have been deposited to the ProteomeXchange Consortium via the PRIDE [71] partner repository with the dataset identifier PXD015518. The RNAseq data have been deposited to the GEO database [72] with the identifier GSE138379.
