# Supplementary material for: Fibroblast‐specific genome‐scale modelling predicts an imbalance in amino acid metabolism in Refsum disease
Source: FEBS J. 2020 Mar 31;287(23):5096–113. doi: 10.1111/febs.15292 (PMC7754141; doi:10.1111/febs.15292)
Supplement: Supplementary file 1 — Table S1. Manual curation of the model including added and curated reactions [Fixes], deleted reactions [ Recon3D_del], added metabolites [Added_mets], and information about the media constraints [HAM'sF10]. Table S2. Fibroblast specific genes. Table S3. Detailed information about the ATP yields for Figs 3 and 4. Table S4. Genes and proteins significantly changed between RD and CTRL fibroblasts. Table S5. Detailed results of the metabolic tasks analysis in the fibroblast model.Table S6. Comparison between the exchanged amino acids in model prediction and in the in vitro experiments (CTRL + phyt group). For a full list of predicted uptake and secretion rates of metabolites see Table S7. Table S7. Detailed comparison at the level of secretion and uptake reactions between healthy and Refsum models forced to take up phytanate. Fig. S1. Additional data on amino acid uptake and secretion rates in the fibroblast CTRL (n = 6; green) and RD (n = 5; blue) cultures exposed to phytol for 96 h (shown as mean ± SD). Rates were calculated based on the fresh medium measurements. Fig. S2. Additional experimental data of (A) amino acids and (B) glucose determinations in the medium of the fibroblast CTRL (n = 6) and RD (n = 5) cells after incubation at indicated time points (shown as mean ± SD). For details, see Fig. 6C. (C) Protein concentrations of cell pellets after incubation at indicated time points. (D) Growth curves of attached cells for the indicated time points (left panel), and statistical analysis of the total area under the curve per cell line after 7 days of incubation (right panel). Data are shown as bar plots. [file FEBS-287-5096-s001.zip › febs15292-sup-0005-Supinfo.pdf]

FEBS PRESS science publishing by scientists
WILEY
The FEBS Journal
www.febsjournal.org

Fibroblast-specific genome-scale modelling predicts an imbalance in amino acid metabolism in Refsum disease

Agnieszka B. Wegrzyn, Katharina Herzog, Albert Gerding, Marcel Kwiatkowski, Justina C. Wolters, Amalia M. Dolga, Alida E. M. van Lint, Ronald J. A. Wanders, Hans R. Waterham and Barbara M. Bakker

DOI: 10.1111/febs.15292

Supplementary Information

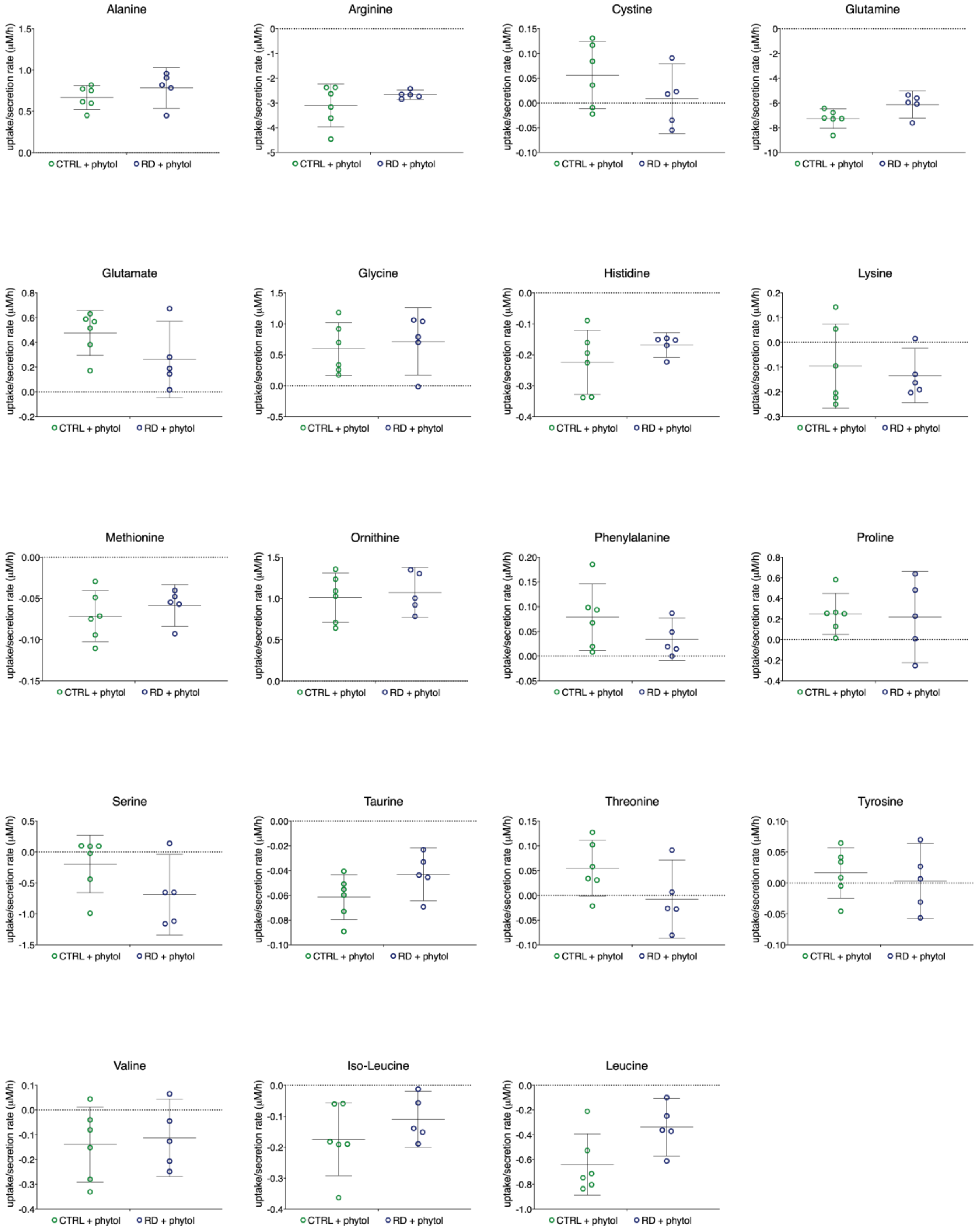

Figure S1. Additional data on amino acid uptake and secretion rates in the fibroblast CTRL (n=6; green) and RD (n=5; blue) cultures exposed to phytol for 96 hours (shown as mean +/- SD). Rates were calculated based on the fresh medium measurements.

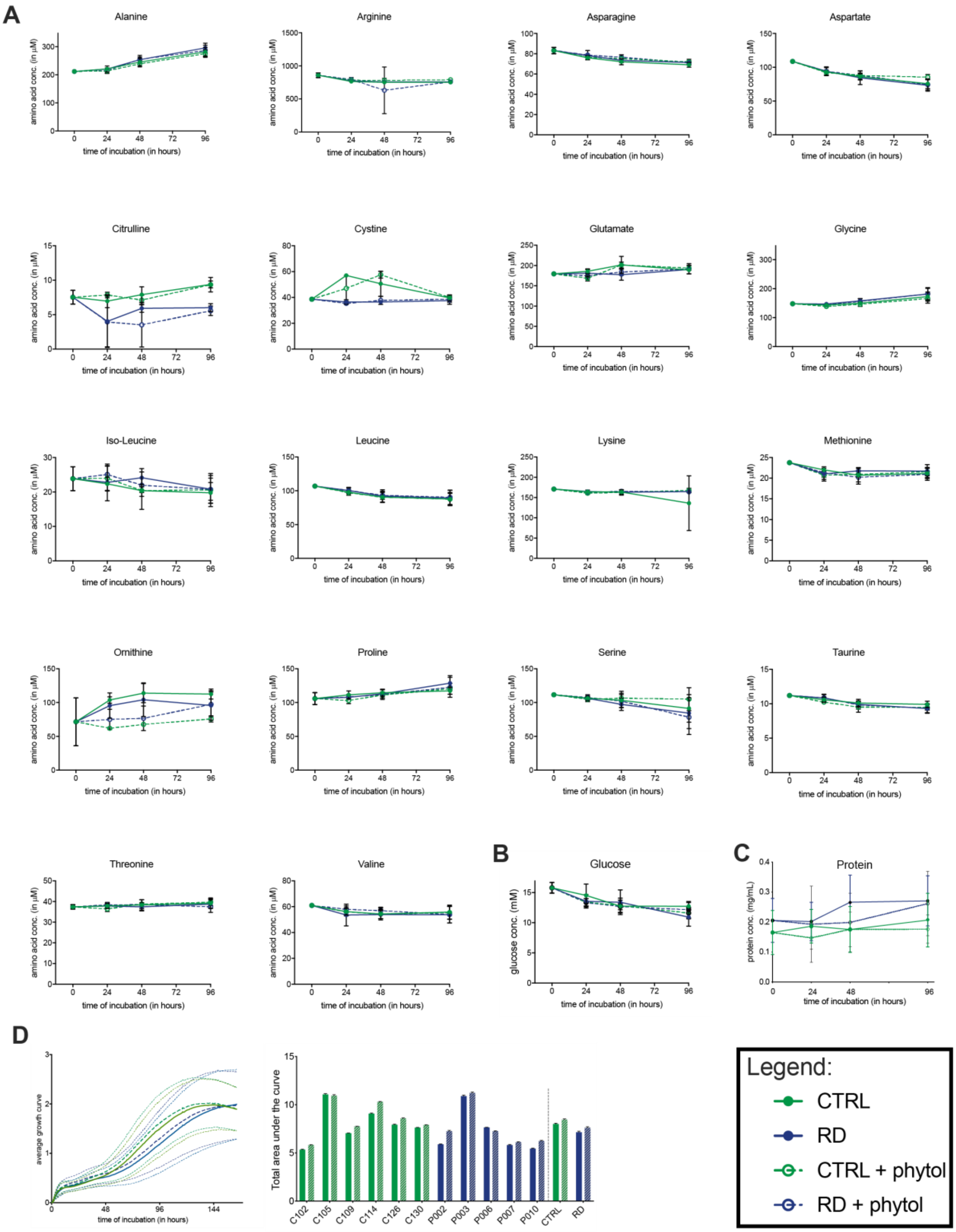

Figure S2. Additional experimental data of A) amino acids and B) glucose determinations in the medium of the fibroblast CTRL (n=6) and RD (n=5) cells after incubation at indicated time points (shown as mean +/- SD). For details, see Fig. 6C. C) Protein concentrations of cell pellets after incubation at indicated time points. D) Growth curves of attached cells for the indicated time points (left panel), and statistical analysis of the total area under the curve per cell line after 7 days of incubation (right panel). Data are shown as bar plots.

Supplementary Table 3. Model predictions of ATP yields from a single carbon source without and with added glutathione (see also Figure 3 and 4).

Carbon_source	modelR3D_FAD	modelR3D_X_c	fibroblastC	fibroblastR	Supplement	modelR3D_FAD	modelR3D_X_c	fibroblastC	fibroblastR
EX_glc_D[e]	32,00	32,00	32,00	32,00	EX_gthrd[e]	80,00	80,00	79,49	79,49
EX_fru[e]	32,00	32,00	0,00	0,00		80,00	80,00	47,50	47,50
EX_lac_L[e]	15,00	15,00	0,00	0,00		63,00	63,00	47,50	47,50
EX_but[e]	22,00	22,00	22,00	22,00		70,00	70,00	69,49	69,49
EX_caproic[e]	34,50	37,00	37,00	37,00		82,50	85,00	84,49	84,49
EX_octa[e]	52,00	52,00	52,00	52,00		100,00	100,00	99,49	99,49
EX_dca[e]	67,00	67,00	66,99	66,99		115,00	115,00	114,49	114,49
EX_ddca[e]	82,00	82,00	81,99	81,99		130,00	130,00	129,49	129,49
EX_ttdca[e]	97,00	97,00	96,99	96,99		145,00	145,00	144,49	144,49
EX_hdca[e]	113,00	113,00	111,99	111,99		161,00	161,00	159,49	159,49
EX_ocdca[e]	129,00	129,00	127,99	127,99		177,00	177,00	175,49	175,49
EX_arach[e]	143,00	143,00	141,99	141,99		191,00	191,00	189,49	189,49
EX_docosac[e]	157,00	157,00	155,99	155,99		205,00	205,00	203,48	203,48
EX_lgnc[e]	168,00	168,00	165,99	165,99		216,00	216,00	213,48	213,48
EX_hexc[e]	178,50	178,50	177,49	177,49		226,50	226,50	224,98	224,98
EX_phyt[e]	-1,00	68,50	61,65	38,80		46,50	116,50	109,30	86,46
EX_ala_L[e]	15,00	15,00	15,00	15,00		63,00	63,00	62,50	62,50
EX_arg_L[e]	27,50	27,50	7,50	7,50		75,50	75,50	55,50	55,50
EX_asn_L[e]	15,00	15,00	-1,00	-1,00		63,00	63,00	37,50	37,50
EX_asp_L[e]	15,00	15,00	15,00	15,00		63,00	63,00	62,50	62,50
EX_cys_L[e]	17,50	17,50	17,50	17,50		65,50	65,50	64,99	64,99
EX_gln_L[e]	22,50	22,50	22,50	22,50		70,50	70,50	69,99	69,99
EX_glu_L[e]	22,50	22,50	22,50	22,50		70,50	70,50	69,99	69,99
EX_gly[e]	8,00	8,00	7,50	7,50		56,00	56,00	55,00	55,00
EX_his_L[e]	25,50	25,50	0,50	0,50		73,50	73,50	49,00	49,00
EX_ile_L[e]	19,00	19,00	17,75	17,75		67,00	67,00	65,25	65,25
EX_leu_L[e]	33,50	33,50	33,50	33,50		81,50	81,50	80,99	80,99

Carbon_source	modelR3D_FAD	modelR3D_X_c	fibroblastC	fibroblastR	Supplement	modelR3D_FAD	modelR3D_X_c	fibroblastC	fibroblastR
EX_lys_L[e]	32,00	32,00	32,00	32,00		80,00	80,00	79,49	79,49
EX_met_L[e]	11,50	11,50	9,75	9,75		59,50	59,50	58,25	58,25
EX_phe_L[e]	33,00	33,00	2,50	2,50		81,00	81,00	50,00	50,00
EX_pro_L[e]	26,50	26,50	21,50	21,50		74,50	74,50	68,99	68,99
EX_ser_L[e]	13,50	13,50	12,50	12,50		61,50	61,50	60,00	60,00
EX_thr_L[e]	18,50	18,50	1,25	1,25		66,50	66,50	48,75	48,75
EX_trp_L[e]	39,50	39,50	17,50	17,50		87,50	87,50	64,99	64,99
EX_tyr_L[e]	35,50	35,50	2,50	2,50		83,50	83,50	50,00	50,00
EX_val_L[e]	29,00	29,00	29,00	29,00		77,00	77,00	76,49	76,49
EX_sarcs[e]	15,00	15,00	13,00	13,00		63,00	63,00	60,50	60,50

Supplementary Table 4. Differentially expressed genes and proteins; FC – fold change (Refsum vs Control)

Gene	Name and function	log2(FC)	adj. p-value
PODXL	podocalyxin like; cell cycle	1,79	1,7E-09
ARK1	aurora kinase A; cell cycle	1,70	2,8E-08
KIF20A	kinesin family member 20A; cell cycle	1,67	7,4E-07
ZNF367	zinc finger protein 367; cell cycle	1,65	3,2E-06
BIRC5	baculoviral IAP repeat containing 5; cell cycle (neurons), protein ubiquitination	1,59	1,5E-05
APOBEC3B	apolipoprotein B mRNA editing enzyme catalytic subunit 3B; cell adhesion & migration	1,58	1,5E-05
IQGAP3	IQ motif containing GTPase activating protein 3; transcriptional activator	1,56	1,6E-05
KIFC1	kinesin family member C1; cell cycle	1,56	2,0E-05
CENPM	centromere protein M; DNA deaminase	1,53	3,0E-05
PLK1	polo like kinase 1; GTPase activity	1,50	3,8E-05
CDC20	cell division cycle 20; spindle formation	1,50	3,8E-05
TK1	thymidine kinase; higher in proliferating cells	1,17	6,7E-04
**Protein**	**Name and function**	**log2(FC)**	**p-value**
PDLIM1	PDZ and LIM domain protein 1; structural; cytoskeletal protein that may act as an adapter that brings other proteins (like kinases) to the cytoskeleton	1,76	1,0E-03
HEMO	Hemopexin; heme binding and transport in plasma (no clue why it's here then)	1,59	3,0E-03
DCD	Dermcidin; antimicrobial; displays antimicrobial activity thereby limiting skin infection by potential pathogens in the first few hours after bacterial colonization	1,5	3,0E-03
CASPE	Caspase-14; epidermal differentiation, regulates maturation by proteolytically processing filaggrin	1,49	2,0E-03
SPR2A	Small proline-rich protein 2A; structural; Cross-linked envelope protein of keratinocytes.	1,45	9,0E-03
FILA2	Filaggrin-2; structural; Intermediate filament-associated and psoriasis-susceptibility protein	1,34	1,0E-03
FILA	Filaggrin; structural; an intermediate filament-associated protein that aggregates keratin intermediate filaments in mammalian epidermis	1,22	0,0E+00
ARMT1	Protein-glutamate O-methyltransferase; methyltransferase; Methylates glutamate residues of target proteins to form gamma-glutamyl methyl ester residues. Methylates PCNA, suggesting it is involved in the DNA damage response	1,15	5,0E-03
K2C80	Keratin, type II cytoskeletal 80; structural	1,12	2,0E-03
PLAK	Junction plakoglobin; structural; cell adhesion	1,1	1,0E-03
DSC1	Desmocollin-1; structural, cell-cell adhesion;	1,07	1,0E-02
DSG1	Desmoglein-1; structural, cell-cell adhesion;	1,04	9,0E-03
K1C10	Keratin, type I cytoskeletal 10; structural	1,02	2,0E-03

BGH3	Transforming growth factor-beta-induced protein ig-h3; cell adhesion	1,02	7,0E-03
K2C5	Keratin, type II cytoskeletal 5; structural	1,01	2,0E-03
KPCA	Protein kinase C alpha type; cell proliferation, apoptosis, differentiation, migration and adhesion	-1,09	9,0E-03
DDX24	ATP-dependent RNA helicase DDX24; ATP-dependent RNA helicase	-1,14	8,0E-03
CA198	Uncharacterized protein C1orf198; orphan	-1,42	6,0E-03
**Proteins with peptides detected in proteomics but no RNA detected in transcriptomics**			
PI4KA	Phosphatidylinositol 4-kinase alpha		
AL1L1	Cytosolic 10-formyltetrahydrofolate dehydrogenase		
GMPPB	Mannose-1-phosphate guanyl transferase beta		
AOC3	Membrane primary amine oxidase		
NDKB	Nucleoside diphosphate kinase B		
AK1D1	3-oxo-5-beta-steroid 4-dehydrogenase		

Supplementary Table 6. Comparison between the exchanged amino acids in model prediction and in the *in vitro* experiments (CTRL + phyt group). For a full list of predicted uptake and secretion rates of metabolites see Supplementary Table 7.

	CTRL + phytol	predicted by the model	match
Alanine	secreted	uptaken	NO
Arginine	uptaken	uptaken	YES
Asparagine	uptaken	uptaken	YES
Aspartate	uptaken	uptaken	YES
Citrulline	secreted	secreted	YES
Cysteine	secreted	secreted	YES
Glutamine	uptaken	uptaken	YES
Glutamate	secreted	secreted	YES
Glycine	secreted	secreted	YES
Histidine	uptaken	uptaken	YES
Isoleucine	uptaken	uptaken	YES
Leucine	uptaken	uptaken	YES
Lysine	uptaken	uptaken	YES
Methionine	uptaken	uptaken	YES
Phenylalanine	secreted	uptaken	NO
Proline	secreted	uptaken	NO
Serine	uptaken	uptaken	YES
Taurine	uptaken	secreted	NO
Threonine	secreted	uptaken	NO
Tyrosine	secreted	uptaken	NO
Valine	uptaken	uptaken	YES
Sarcosine	uptaken	uptaken	YES
correct			73%
incorrect			27%
